# Can Bibliographic Pointers for Known Biological Data Be Found Automatically? Protein Interactions as a Case Study

**DOI:** 10.1002/cfg.91

**Published:** 2001-08

**Authors:** Christian Blaschke, Alfonso Valencia

**Affiliations:** Protein Design Group National Centre for Biotechnology CNB-CSIC Cantoblanco Madrid E-28049 Spain

## Abstract

*The Dictionary of Interacting Proteins* (DIP) (Xenarios *et al.*, 2000) is a large repository of protein interactions: its March 2000 release included 2379 protein pairs whose interactions have been detected by experimental methods. Even if many of these correspond to poorly characterized proteins, the result of massive yeast two-hybrid screenings, as many as 851 correspond to interactions detected using direct biochemical methods.

We used information retrieval technology to search automatically for sentences in Medline abstracts that support these 851 DIP interactions. Surprisingly, we found correspondence between DIP protein pairs and Medline sentences describing their interactions in only 30% of the cases. This low coverage has interesting consequences regarding the quality of annotations (references) introduced in the database and the limitations of the application of information extraction (IE) technology to Molecular Biology. It is clear that the limitation of analyzing abstracts rather than full papers and the lack of standard protein names are difficulties of considerably more importance than the limitations of the IE methodology employed. A positive finding is the capacity of the IE system to identify new relations between proteins, even in a set of proteins previously characterized by human experts. These identifications are made with a considerable degree of precision.

This is, to our knowledge, the first large scale assessment of IE capacity to detect previously known interactions: we thus propose the use of the DIP data set as a biological reference to benchmark IE systems.


**Comparative and Functional Genomics**
*Comp Funct Genom* 2001; **2**: 196–206.
**DOI:** 10.1002/cfg.91

**Research Article**

### Can bibliographic pointers for known biological data be found automatically? Protein interactions as a case study

Christian Blaschke and Alfonso Valencia*
*Protein Design Group, National Centre for Biotechnology, CNB-CSIC, Cantoblanco, Madrid E-28049, Spain*

*Correspondence to: A. Valencia, Protein Design Group, National Centre for Biotechnology, CNB-CSIC, Cantoblanco, Madrid E-28049, Spain. E-mail: valencia@cnb.uam.es

Received: 27 March 2001
Accepted: 25 June 2001

### Abstract

***The Dictionary of Interacting Proteins*** **(DIP) (Xenarios *et al.*, 2000) is a large repository of protein interactions: its March 2000 release included 2379 protein pairs whose interactions have been detected by experimental methods. Even if many of these correspond to poorly characterized proteins, the result of massive yeast two-hybrid screenings, as many as 851 correspond to interactions detected using direct biochemical methods.**

**We used information retrieval technology to search automatically for sentences in Medline abstracts that support these 851 DIP interactions. Surprisingly, we found correspondence between DIP protein pairs and Medline sentences describing their interactions in only 30% of the cases. This low coverage has interesting consequences regarding the quality of annotations (references) introduced in the database and the limitations of the application of information extraction (IE) technology to Molecular Biology. It is clear that the limitation of analyzing abstracts rather than full papers and the lack of standard protein names are difficulties of considerably more importance than the limitations of the IE methodology employed. A positive finding is the capacity of the IE system to identify new relations between proteins, even in a set of proteins previously characterized by human experts. These identifications are made with a considerable degree of precision.**

**This is, to our knowledge, the first large scale assessment of IE capacity to detect previously known interactions: we thus propose the use of the DIP data set as a biological reference to benchmark IE systems. Copyright © 2001 John Wiley & Sons, Ltd.**

**Keywords:** **proteomics; information extraction; protein interactions; yeast two-hybrid; databases**

### Introduction

Proteomics technology is delivering large sets of information on protein interactions, in quantities unprecedented in classical molecular biology (Rain *et al.*, 2001, Fromont-Racine *et al.*, 1997, Ito *et al.*, 2000, Schwikowski *et al.*, 2000). Several initiatives are underway to design databases able to store and manipulate this new information (Eilbeck *et al.*, 1999, Xenarios *et al.*, 2000, Bader *et al.*, 2001). Interaction databases are becoming essential tools in molecular biology research, since they will provide the basis for integrating functional genomic and proteomic data (*Nature* Supplement 2000).

Databases for the storage of protein interactions may also provide gold standards for prediction methods, such as those based on genomic information (Eisenberg *et al.*, 2000), or on extracting information from the scientific literature (Blaschke *et al.*, 1999, Rindflesch *et al.*, 1999, Rindflesch *et al.*, 2000, Proux *et al.*, 2000, Sekimizu *et al.*, 1998, Thomas *et al.*, 2000).

The quality of the interaction databases, as in the case of the sequence databases, depends not only on the quality of the information stored, but also on the ability to trace the origin of the information. For historical and practical reasons, these links were only partially incorporated in the databases.

Copyright © 2001 John Wiley & Sons, Ltd.

SWISS-PROT has recently launched efforts to link database features with the corresponding references and pointers in a detailed manner (R. Apweiler, personal communication).

The Database of Interacting Proteins (Xenarios *et al.*, 2000) is a good example of an up-to-date repository dedicated to storage of protein-protein interactions. Each entry contains the names of the two interacting proteins, together with the experimental technique used for describing the interaction, and bibliographic references in the form of Medline pointers. Two other main information repositories on protein interactions are PIR (Barker *et al.*, 2000) and BIND (Bader *et al.*, 2001). PIR contains a catalog of macromolecular complexes, whereas BIND has information about protein interactions and molecular complexes (1332 interactions, 41 molecular complexes), plus additional information on interactions with DNA, RNA, and other molecules. Only a limited number of PIR and BIND entries are linked to bibliographic references.

It is this early phase of development in which it is important to assess the extent to which database entries are adequately linked to bibliographic references, and when it is of interest to determine the utility of developing tools to aid in the data annotation process. DIP is an interesting case for such studies because it is hand curated and provides links to the literature used to deduce each interaction.

Employing an updated version of our information extraction (IE) system (Blaschke *et al.*, 1999), we found the correct evidence in the literature for less than a third of the DIP interactions. The main reasons for the low recall rate were inconsistency in protein nomenclature and the lack of relevant information in the abstracts. Application of automatic information retrieval tools detected new interactions between proteins described in DIP as participants in other interactions. This observation indicates the potential of information retrieval tools as aids during the construction of interaction databases.

### Methods

### DIP in numbers

DIP database entries (Xenarios *et al.*, 2000) were downloaded as a flat file from **http://dip.doe-mbi.ucla.edu** as deposited by March 8 2000.

The DIP entries can be divided into two categories, those taken directly from yeast two-hybrid (y2h) screenings and those characterized by more classical methods. While the y2h pairs correspond mainly to proteins of unknown function, the interactions described using 'classical' experimental techniques are considered to demonstrate more reliable interactions between well-characterized proteins (Table 1).

It is from this latter group (the 'classical' experiments) that links are found from DIP entries to publications, in the form of pointers to Medline abstracts. This set contains approximately one different reference for each interaction, whereas in the y2h interaction set, hundreds of interactions are linked to a few Medline references. The work described here was carried out mainly with the 'classical' experiment set of interactions, except where explicitly stated.

In DIP, each protein is identified by an ID number, a name, and pointers to different sequence databases; PIR (Barker *et al.*, 2000), SWISS-PROT (Bairoch and Apweiler 2000), GenBank (Benson *et al.*, 1998), and PDB (Sussman *et al.*, 1998), when available. For example, the full set of 2149 proteins contains 1542 links to SWISS-PROT.

### Detection of protein names

The absence of naming conventions for proteins makes automated detection of similarities between protein names difficult. Protein names are subject to many variations, including changes over time, writing variants, in particular names of mutated proteins and alleles. In addition specific and general names are used simultaneously: for example, sequence databases tend to use precise technical names, such as 'cyclin-dependent kinase inhibitor p27', whereas common names are often used in the literature, e.g. 'p27kip1'. The identification of protein names thus poses a difficult problem.

To detect as many proteins as possible, DIP protein names were enriched with synonyms extracted from the SWISS-PROT database (Figure 1). Alternative names were identified in the description (DE field) and the gene names lines (GN field). Using this procedure, the initial set of 1542 DIP protein names (the fraction of the 2149 proteins in DIP containing a pointer to SWISS-PROT) was supplemented with 7164 to reach a total of 8706 forms, including synonyms and spelling variants. Some variations were allowed to increase the possibility of matching related names, such as IL 6 with IL6 and

Copyright © 2001 John Wiley & Sons, Ltd. 

Table 1. Information on proteins and interactions in DIP

| Information source | DIP entries (n°) | Protein names (n°) | Medlines linked to DIP entries (n°) | SWISS-PROT linked to DIP entries (n°) |
|---|---|---|---|---|
| Classical experiments | 851 | 827 | 427 | nd |
| y2h | 1528 | 1466 | 105 | nd |
| Total | 2379 | 2149 (1) | 514 (1) | 1542 |

nd: numbers not calculated.
(1) some protein interactions have been described by both classical and y2h: the corresponding DIP entries include references to both type of experiments.

IL-6, ste18 with ste18p (rule [name]p), erbb2 will match p185erbb2 (rule p[number][name]).

Some names for different proteins in DIP are so similar that reliable distinction between them became impossible, e.g., p52shc and p52(Shc) are a mouse and a human protein that form part of different interactions in DIP. Our automated system would consider them identical, which raises several questions: Should these two entries be treated as identical? If not, how could they be differentiated? How many other such instances are there, and what would be the consequences of treating them as identical?

### Collecting the text corpus

Initially, we used the small collection of 514 Medline abstracts quoted directly in DIP. A larger

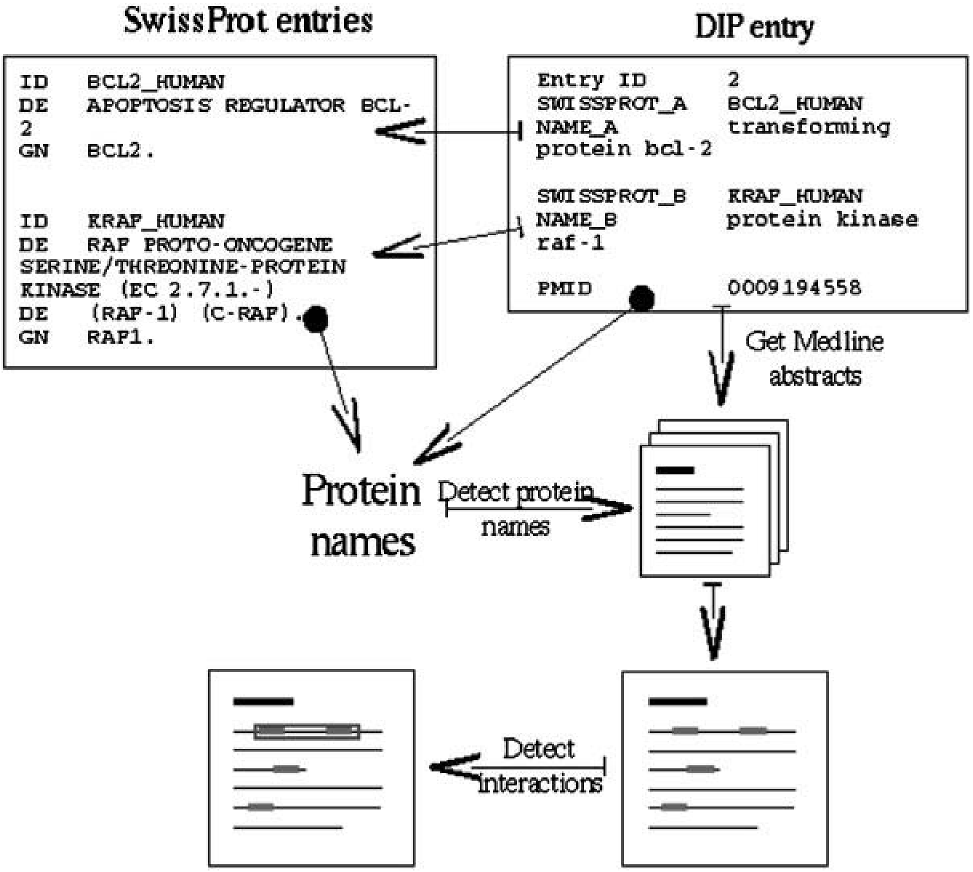

**Figure 1.** Creation of the protein name list corpus of abstracts was subsequently collected by direct search of PubMed (PubMed 2000) with the protein names and synonyms; this second collection contains 61,323 Medline entries.

### Extraction of the interactions

To extract interactions, an extension of our previously implemented system (Blaschke *et al.*, 1999) was used. This system is based on matching sentences from the text corpus with predefined rules (known as patterns or frames). We have collected eight main rules with several subrules. The first of them is the most informative one.

The current list of rules includes:

(i) protein [word]* [verb] [word]* protein
(ii) [verb] of [word]* protein [word]* [by,to] [word]* protein
(iii) [noun] of [word]* protein [word]* [by,with] [word]* protein
(iv) [noun] between [word]* protein [word]* and [word]* protein
(v) protein [word]* protein [word]* [complex/es, dimer, heterodimer]
(vi) complex formed between [word]* protein [word]* and [word]* protein
(vii) complex/es of [word]* protein [word]* and [word]* protein
(viii) protein [word]* forms a complex with [word]* protein

Sub-rules are used to incorporate specific cases. For example, negative forms of the rules are encoded as sub-rules. In the case of rule 1, the following instances have been incorporated:

(i) protein [word]* [verb] [word]* but not [word]* protein
(ii) protein [word]* cannot [word]* [verb] [word]* protein
(iii) protein [word]* does not [word]* [verb] [word]* protein
(iv) protein [word]* did not [word]* [verb] [word]* protein
(v) protein [word]* was not [word]* [verb] [word]* protein
(vi) protein [word]* not [word]* [verb] [word]* by [word]* protein
(vii) protein [word]* not required for [word]* [verb] [word]* protein
(viii) protein [word]* failed to [word]* [verb] [word]* protein

The nouns and verbs are taken from a hand constructed list containing nouns such as *activation*, *phosphorylation* or *interaction*, and verbs such as *activates*, *binds* or *phosphorylates*. Rules are applied directly to the text by string comparison. We have not yet found a suitable parsing strategy that could render similar results in real scenarios.

The number of intervening words ([word]*) is used as part of a scoring system that favors shorter sentences in which protein names are closer together. The final scores are additionally modified to consider the number of sentences (and Medline abstracts) in which each interaction is observed.

In discussion of possible error sources, it must be noted that the IE system does not differentiate direct physical interactions from all other possible relations. The typical construction 'protein A activates protein B' does not necessarily imply direct inter-protein contact, as it can be mediated by a third protein. The sentence 'The expressed **p53** protein showed nuclear localization and its expression was associated with an induction of **p21** and **bax** expression' relates **p53** with **p21** and **bax** but does not imply a physical interaction between them.

The final interaction network is presented in a graphic user interface that enables manipulation and visualization of the system, analysis of the individual relationship, and retrieval of the sentences and abstracts used to deduce the relationship. This graphic interface was also useful for manual checking of hundreds of interactions (an on-line version showing the analysis of various biological systems is accessible at: **http://www.pdg.cnb.uam.es/suiseki/**).

### Results

### Linking Medline sentences to DIP entries

We initially evaluated the number of interactions that can be retrieved automatically from the Medline abstracts quoted directly in the corresponding DIP entries. For 210 entries, approximately 25% of the 851 interactions reported in DIP, the system finds the corresponding links; that is, the information extraction system identified sentences containing the two protein names of a DIP entry in a sentence determined by the rules as indicative of interaction. These cases are the basis for the automatic assignment of DIP entries to the basic

Copyright © 2001 John Wiley & Sons, Ltd. 

information coded in the corresponding scientific text.

Examples of correct identification could be:

'The Cdc2 protein kinase controls **Cdc10/Sct1 complex** formation' which includes information about the complex of **Cdc10** with **Sct1** (DIP entry 332).

'**p27Kip1** binds the complex as an extended structure **interacting with** both **cyclin A** and **Cdk2**′ for the interaction of **p27Kip1** with **cyclin A** and **Ddk2** (DIP entry 550).

'In addition, **JAK2 phosphorylated Raf-1** at sites different from those phosphorylated by pp60v-src', from where the interaction of **JAK2** with **Raf-1** was deduced (DIP entry 600).

### Assessing the accuracy of detected interactions

Several factors contribute to the surprisingly low recall rate, making detection of Medline sentences impossible for 75% of the DIP entries. First, information about the interactions is not included in many of the abstracts, obviously rendering them undetectable. Second, in many cases the DIP protein (gene) names or their synonyms were not found in the corresponding text sources. Finally the information was not extracted in some cases because the description of the interactions in the text is too complex for the relatively simple rules implemented in the extraction system.

Detailed analysis of 100 random examples (Table 2) of failed detection indicates that the identification of protein names remains the most serious problem. Second in order of importance is that information about interactions is not always explicitly given in the abstracts. Surprisingly, the limitations of the information extraction system appears to be the least crucial factor.

### Text corpus coverage

In approximately one third of the cases, the information on the interactions was not found in the abstract. In the cases checked manually, this information was found in the text of the articles, mainly in the results section. In some cases the information was given in tables, rendering automatic retrieval even more difficult.

Table 2. Reasons for non-detection of interactions in the manual analysis of 100 random examples

| Reason for failure | % |
|---|---|
| Information not in abstract | 35% |
| Names not correctly detected | 44% |
| Information extraction system failed | 21% |

### Detection of protein names

Problems arise here because protein names, contrary to chemical compounds, are not standardized and are often used in different forms in free text style. The lack of unique forms complicates detection and matching of names between different text sources, or text sources and databases. Some examples illustrate different forms of this problem:

In the following sentence, the names are expressed in a complex form easy to identify by a human, but which presents difficulties to an automatic system. 'The replacement of Thr161, a residue conserved and phosphorylated in other protein kinases, with valine inhibits cdc2 association with A and B cyclins'. This sentence is taken from an abstract linked to the DIP interaction between *cdc2* with *cyclin A* and *cyclin B*. The cyclin A name is not reconstructed correctly by our IE system, and the list of synonyms cannot include A as a synonym of cyclin A.

In other cases, the relationship between synonyms and protein names cannot be identified. One synonym of *transcription factor 3* is *immunoglobulin enhancer binding factor e12*, as extracted from the information in SWISS-PROT. But in sentences like 'Although several lines of evidence suggest that MRF4 and E12 or myogenin and E12 hetero-oligomers exist,...' *E12* is used as a short form of the name, making matching very difficult for the automatic system.

An example of mismatch between similar names that are taken as different by the automatic system is the difference between *cdc25* and *cyclin B* and the more specific forms *cdc25a* and *Cyclin B1* in the sentence 'The motif may represent an activating domain that has to be provided to cdc25a by intermolecular interaction with cyclin B1'. Whether or not *cdc25* and *cyclin B are* synonyms of these proteins is an impossible decision for the automatic system without the aid of explicit references in the synonym list.

Some synonyms tend to be too general or too ambiguous. This is the case of some SWISS-PROT entries that provide very general protein classes as protein names, contributing to the overlap between unrelated proteins when interpreted as synonyms of

Copyright © 2001 John Wiley & Sons, Ltd. 

other, more specific protein names. The description line of the protein PCNA in SWISS-PROT is 'PROLIFERATING CELL NUCLEAR ANTIGEN (PCNA) (CYCLIN)', where PCNA is the name given in the Gene name field. During building of the list of synonyms, the gene name (PCNA) and the protein name (CYCLIN) would be considered synonyms by our system, creating a single node in the interaction network that would attribute to PCNA all the many interactions known for cyclins. As many of these cases as possible were removed manually from the synonym list.

#### Limitations of the information extraction methodology

Even in the cases in which protein names are correctly identified in the abstracts, further errors can be introduced due to the simple approach adopted by the information extraction system. This occurs when the system fails to match the sentence to its internal rules, missing the connection between the proteins. Difficult sentences, complex syntactic constructions and implicit information generally pose problems for the simple rules implemented in current systems.

An example for which information cannot be extracted because it is implicit in the previous sentence is the interaction between *SNF1* and *SIP1* in 'A genetic method, the two-hybrid system, was used to identify four genes encoding proteins that interact with the SNF1 protein kinase from yeast. One of the genes, SIP1, was independently isolated as a multi-copy suppressor ..'.

Current rules are insufficient for capturing the interaction in an example such as 'We used the two-hybrid system to demonstrate that SIR4 can form homodimers', where the relationship defined by being part of a homodimer is not previously described with a rule in our IE system.

In many cases, the constructions are far too complex for the system. One such example is the sentence 'Gel retardation assays demonstrated that fos B protein positively influences the binding of c-jun and jun B proteins to an AP-1 binding consensus sequence, suggesting that fos B protein plays a role in control of gene expression'. In this case *AP-1* is a transcription factor and the sentence means that *fos B* binds to the same DNA sequence as the transcription factor *AP-1*, and not that *fos B* binds to *AP-1*, as the system will wrongly interpret.

Another example is provided by the sentence 'The observation that IL-3 interacts with receptors for GM-CSF and IL-5 may have a bearing on its stronger functional effects and suggests a major role for IL-3 in the pathogenesis of hypersensitivity syndromes'. In this sentence *GM-CSF* and *GM-CSF receptor* are confused by the IE system, which will wrongly deduce an interaction between *IL-3* and *GM-CSF*.

Manual inspection of a representative set of cases reveals that the IE system incurs this type of error in less than 10% of the cases (Table 3).

### Extending the search to the entire Medline

The text corpus was extended with 61,323 additional Medline entries containing at least two of the protein names quoted in the 851 DIP interactions. In this larger set, additional sentences were found for interactions in 378 of the 851 cases (210 previously observed and 98 new interactions, Table 3). More than one abstract often contained the same information: thus, more than 4 abstracts represented the same interaction in 172 cases. The drawback to this analysis is that no information was found for as many as 592 interactions, even when all Medlines were inspected, pointing again to the limitations of the Medline information.

In its favor, this system identified sentences for 98 DIP interactions that were impossible to match when only the Medline referenced in DIP was used (Table 3). Manual evaluation showed that 70% of these identifications are correct; that is, 70% are sentences linking two protein names with a valid construction that indicates interaction. The lower success rate compared with the set of Medlines selected by the human experts behind DIP is predictable, as the larger corpus is not curated and may contain irrelevant abstracts.

Table 3. Automatically detected interactions and manual assessment of their accuracy

| Corpus | Automatically detected DIP interactions (n°) | Correctly detected | % |
|---|---|---|---|
| 514 Medlines directly linked to DIP | 210 | 190 | 90.5% |
| 61,323 Medlines containing DIP protein names | 98 new (308 total) | 69 new (259 total) | 70.5% |

Copyright © 2001 John Wiley & Sons, Ltd.

### Exploring new interactions discovered by the IE system

Further searches were carried out to detect interactions between DIP proteins using different corpora.

In the small corpus composed of 514 Medline abstracts directly linked to the DIP, we found 335 new interactions not quoted in DIP. This shows that even some information (for DIP entries) is not contained in the abstracts, additional information that may have escaped the attention of human annotators can be extracted. A total of 206 correct constructions were counted, 61% accuracy (Table 4).

An example of correct detection is the interaction between *PCNA* and *cdk2*, deduced from the sentence: 'Polymorphic cell nuclear antigen (PCNA) protein bound to cdk2 was a better indicator for cell proliferation and cdk2 kinase activity than the PCNA labelling index', which is not included in DIP.

Inspection of the large corpus of 61,323 Medline abstracts revealed 1940 potential new interactions between DIP proteins. All were not analyzed, but a rough estimate indicates between 30 and 50% correct identifications.

### References for interactions detected by the yeast 2-hybrid system

The DIP includes a large set of interactions that were described based on massive yeast two-hybrid screenings. These interactions are linked in DIP only to papers describing the yeast two-hybrid experiments. For some, Medline references were recovered corresponding to the identification of the same interactions.

With a level of more than 70% correct detection Medline sentences were found for 106 interactions, 45 in the Medline abstracts linked to DIP and the remainder in other Medline abstracts (Table 5).

Although in numerical terms these interactions constitute a small fraction of the 1798 interactions listed in DIP, we believe they provide a good example of the possibilities offered by the IE tools in annotating known interactions.

Table 4. New interactions detected automatically and assessment of their accuracy

| Corpus | New interactions detected automatically (n°) | Correctly detected | % |
|---|---|---|---|
| 514 Medlines directly linked to DIP | 335 | 206 | 61.5% |
| 61,323 Medlines containing DIP protein names | 1940 | not calculated | 30–50% |

### Discussion

Only a fraction of protein interactions that take place in biological systems have been described in the scientific literature (Figure 2). Recent experiments in yeast two-hybrid systems (Ito *et al.*, 2000, Fromont-Racine *et al.*, 1997, Uetz *et al.*, 2000) indicate that the number of known interactions is very small, and that a large number of true interactions has still to be discovered. This can be done by experiments or by computational methods (Eisenberg *et al.*, 2000, Enright *et al.*, 1999, Marcotte *et al.*, 1999, Pellegrini *et al.*, 1999).

The current version of the DIP, one of the first curated databases of protein interactions, covers more than 2000 proteins and 2300 interactions. Most were acquired from recently published large scale y2h experiments (more than 60%), only 40% are based on more precise experimental techniques such as immunoprecipation, X-ray crystallography, gel filtration and ELISA. In addition 68 interactions correspond to indirect genetic experiments.

We explore the possibilities of tracing part of the DIP interactions to their origin in the literature, using an automatic information extraction method. IE extraction is a well established discipline which originated from the early attempts of natural language understanding. The methods in this field are evaluated since the late 80's in the message understanding conferences (MUC). The techniques resulting from the research in this field reach a level of recall and precision of around 80 to 90% when they are properly adapted to the specific domain

Table 5. Interactions automatically detected for DIP interactions extracted from massive yeast 2-hybrid experiments

| Corpus | Interactions (n°) | Correct detections (n°) | % |
|---|---|---|---|
| 514 Medlines directly linked to DIP | 45 | 38 | 84% |
| 61,323 Medlines containing DIP protein names | 61 | 44 | 72% |

Copyright © 2001 John Wiley & Sons, Ltd. 

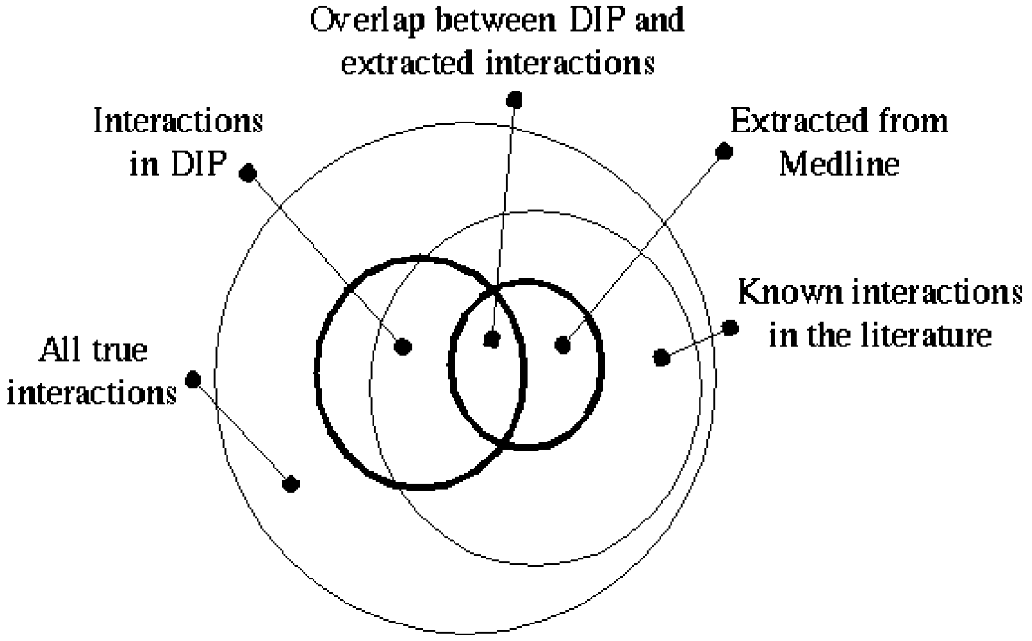

**Figure 2.** Distribution of the protein interaction universe. Of all protein interactions, only a small fraction have been identified and published in the scientific literature. Many more are being discovered with mass screening approaches i.e. yeast two-hybrid systems. A fraction of the known interactions are stored in the DIP database by human experts, corresponding in part to standard biochemical experiments published in the scientific literature and in part to new proteomic data. Current automatic tools can extract some of these interactions from sources such as Medline abstracts. Here we study the relationship between the interactions stored in DIP and automatically-extracted interactions, in an effort to assess how much information in DIP can be traced to its source in Medline sentences knowledge. In the last years a number of attempts were made to apply IE to the problem of protein interactions in biomedical text. In most of the cases a part-of-speech tagger is applied in the first step to get syntactical information of the sentence and grammars are used to get an idea of the meaning of the analyzed sentence. Rindflesch *et al.* (Rindflesch *et al.*, 1999, Rindflesch *et al.*, 2000) detect binding relations between proteins and relations between genes and cells by using external knowledge systems (the UMLS MetaThesaurus from the National Library of Medicine). Different works (Humphreys *et al.*, 2000, Thomas *et al.*, 2000) demonstrated the feasibility of adapting general purpose information extraction systems to the domain of molecular biology in small test cases. Other authors simply concentrate on purely statistical methods counting for example the co-occurrance of protein names or other significant terms in the same abstract without the application of a linguistic methodology (Andrade and Valencia, 1998, Blaschke *et al.*, 2001, Jenssen *et al.*, 2001, Stapley and Benoit, 2000). Other strategies have been used by different authors with varying degrees of success (Proux *et al.*, 2000, Sekimizu *et al.*, 1998, Yakushiji *et al.*, 2001).

With the current state of information extraction technology as applied to molecular biology, it is difficult to compare the capacity of different systems. Most applications have ignored the problem of recognizing protein names, and often evaluate performance on reduced data sets (Proux *et al.*, 2000, Thomas *et al.*, 2000). In the absence of valid standards, it is difficult to determine whether results for controlled scenarios will extrapolate to complex biological situations, such as that described here.

We propose four areas in which our study may have implications.

(i) First, it proposes the DIP data set as "proof of concept" for the possible use of information extraction systems that validate information stored in a database.
(ii) Second, it shows how IE can be used to aid in the database creation process by proposing possible interactions to human annotators. IE technology

Copyright © 2001 John Wiley & Sons, Ltd. 

may thus help overcome the limitations of the historical sequence databases by including accurate references to different database objects in the early phase of their development.

(iii) Third, it allows possibly the first direct evaluation of how much information in an annotated database can be traced to the underlying evidence. Our results indicate that these links can be established only for a small fraction of the entries.

(iv) Finally, we propose the use of the DIP set of interactions as a reference set for different IE technologies. The results presented here could be seen as a base line. The resulting sentences for each DIP entry can be accessed online (**http://www.pdg.cnb.uam.es/blaschke/DIP_analysis.html**). This realistic and biologically relevant set of interactions is probably a better reference point than the partial analysis of small sets of sentences that have characterized previous approaches.

### Is the information available sufficient to link DIP entries to Medline sentences?

The initial phase of this study was to detect DIP protein names in Medline. Given the lack of standard protein names, their identification remains one of the most challenging problems in this field (see discussion by Fukuda *et al.*, 1998 and Proux *et al.*, 1998). Even after extending protein names with synonyms (i.e. alternative names used in SWISS-PROT), only a fraction of the DIP protein names could be identified. This clearly speaks for the need to introduce standard protein and gene names. It would also be desirable that DIP and other databases provide direct ways of converting the names they use into the names used in the scientific literature.

As illustrated by examples (see results), the use of the same name for different proteins or the use of general synonyms increases the ambiguity of protein names and complicates their identification. Extending the number of synonyms by including database entries and related Medline entries would increase the coverage of the system, although this will be at the expense of the detection precision, since more ambiguities would be included.

In addition to the problem of identification, we find that a significant number of DIP protein names are not present in the Medline abstracts mentioned in the DIP entries or in any other Medline abstract. It is possible that these names have been included in DIP after detailed reading of the full papers by the database annotators, and they therefore cannot be found in the abstract. This indicates the need for analyzing full textual sources and trusted web repositories, and not only abstracts.

With the name and synonym information abstracts were identified containing the names of the two interacting proteins for 378 of the 851 DIP interactions, indicating that potentially useful abstracts were identified for 44% of the DIP entries. The limitation of analyzing only Medline abstracts, combined with the difficulty in identifying protein names pose the main difficulties to the development of IE strategies. Our analysis indicates that almost 80% of the missing links to DIP are produced either by errors in the identification of protein names or by the lack of information on the interactions in the abstracts. Only 20% of the cases involve inaccuracies of the IE system (Table 1).

When protein names were identified in an abstract, the IE system failed to detect the interaction correctly only in a small number of cases (less than 10% of the cases; Table 3). Although such cases indicate the need to improve the performance of our IE system (possibly by improving the underlying Natural Language Processing system), the real biological problem appears to require improvement in the detection of protein names and analysis of complete text corpus rather than more sophisticated NLP methodology.

### Finding new interactions beyond the DIP information

Application of the automatic information extraction system provided new information not quoted in DIP. For a fraction of the DIP interactions derived directly from y2h experiments (and not quoted in DIP as having been confirmed by other experiments), the corresponding interactions were found in the literature. This observation demonstrates that biological information can be mined efficiently with current tools, in some cases beyond the obvious effort of the human experts during database construction.

In a number of cases, we found new interactions between proteins that were clearly identified as DIP proteins. We analyzed a number of these new interactions, and found that more than 60% correspond to true interactions. It is thus tempting to

Copyright © 2001 John Wiley & Sons, Ltd. 

propose that automatic extraction systems can be a good guide for the detection of interactions. The leads provided by the automatic systems will have the additional advantage of serving as detailed pointers, binding database entries with key Medline sentences.

### Conclusions

The analysis presented here highlights the problems of incomplete text sources and inconsistent protein names as the main difficulties facing the creation of extended protein interaction repositories and the cross-referencing of existing ones, i.e. SWISS-PROT. These observations indicate that recent reports on the performance of different interaction extraction systems underestimate the importance and difficulty of these problems in real world situations. We thus propose DIP as a realistic scenario for the comparison of IE systems, counting on the initial performance of our system, which identified 259 direct links between DIP entries and Medline sentences. Of these, 190 were identified in Medline abstracts quoted directly in DIP, and 69 were retrieved from other Medline abstracts. In total, for 30.5% of the DIP interactions the bibliographic origin was identified.

The possibilities offered by IE systems in the field of database annotation are illustrated by the discovery of about 2000 new interactions. The validation of these new interactions by human experts could speed up the process of database annotation.

### References

Andrade MA, Valencia A. 1998. Automatic extraction of keywords from scientific text: Application to the knowledge domain of protein families. *Bioinformatics* **14**: 600–607.

Bader GD, Donaldson I, Wolting C, Ouellette BFF, Pawson T, Hogue CWV. 2001. BIND-The Biomolecular Interaction Network Database. *Nucleic Acids Res* **29**: 242–245.

Barker WC, Garavelli JS, Huang H, *et al*. 2000. The Protein Information Resource (PIR). *Nucleic Acids Res* **28**: 45–48.

Benson DA, Boguski MS, Lipman DJ, Ostell J, Ouellette BFF. 1998. GenBank. *Nucleic Acids Res* **26**: 1–7.

Bairoch A, Apweiler R. 2000. The SWISS-PROT Protein Sequence Data Bank and its Supplement TrEMBL. *Nucleic Acids Res* **28**: 46–48.

Blaschke C, Andrade MA, Ouzounis C, Valencia A. 1999. Automatic Extraction of Biological Information from Scientific Text: Protein-Protein Interactions. *Proc Int Conf Intell Syst Mol Biol 1999*: 60–67.

Blaschke C, Oliveros JC, Valencia A. 2001. Mining functional information associated to expression arrays. *Funct Integr Genomics* **1**: 256–268.

Chien CT, Bartel PL, Sternglanz R, Fields S. 1991. The two-hybrid system: a method to identify and clone genes for proteins that interact with a protein of interest. *Proc Natl Acad Sci U S A* **88**: 9578–9582.

Eilbeck K, Brass A, Paton N, Hodgman C. 1999. INTERACT: an object oriented protein-protein interaction database. *Proc Int Conf Intell Syst Mol Biol 1999*: 87–94.

Eisenberg D, Marcotte EM, Xenarios I, Yeates TO. 2000. Protein function in the post-genomic era. *Nature* **405**: 823–826.

Enright AJ, Iliopoulos I, Kyrpides NC, Ouzounis CA. 1999. Protein interaction maps for complete genomes based on gene fusion events. *Nature* **402**: 86–88.

Fromont-Racine M, Rain JC, Legrain P. 1997. Toward a functional analysis of the yeast genome through exhaustive two-hybrid screens. *Nat Genet* **16**: 277–282.

Fukuda K, Tsunoda T, Tamura A, Takagi T. 1998. Information Extraction: Identifying Protein Names from Biological Papers. *Pac Symp Biocomput 1998*: 707–718.

Hishiki T, Collier N, Nobata C, *et al*. 1998. *Genome Inform Ser Workshop Genome Inform 1998*; **9**: 81–90.

Humphreys K, Demetriou G, Geizauskas R. 2000. Two Applications of Information Extraction to Biological Science Journal Articles: Enzyme Interactions and Protein Structure. *Pac Symp Biocomput 2000*: 502–513.

Jenssen TK, Lægreid A, Komorowski J, Hovig E. 2001. A literature network of human genes for high-throughput analysis of gene expression. *Nat Genet* **28**: 21–28.

Ito T, Tashiro K, Muta S, *et al*. 2000. Toward a protein-protein interaction map of the budding yeast: A comprehensive system to examine two-hybrid interactions in all possible combinations between the yeast proteins. *Proc Natl Acad Sci U S A* **97**: 1143–1147.

Nature Supplement on Functional Genomics 2000. Vol. 405, no. 6788

Ohta Y, Yamamoto Y, Okazaki T, Uchiyama I, Takagi T. 1997. Automatic Construction of Knowledge Base from Biological Papers. *Proc Int Conf Intell Syst Mol Biol 1997*: 218–225.

Proux D, Rechenmann F, Julliard L, Pillet V, Jacq B. 1998. Detecting Gene Symbols and Names in Biological Texts: a first step toward pertinent Information Extraction. *Genome Inform Ser Workshop Genome Inform 1998*; **9**: 72–80.

Proux D, Rechenmann F, Julliard L. 2000. A Pragmatic Information Extraction Strategy for gathering Data on Genetic Interactions. *Proc Int Conf Intell Syst Mol Biol 2000*: 279–285.

PubMed 2000. PubMed database at the National Library of Medicine: **http://www.ncbi.nlm.nih.gov**

Rain J, Selig L, De Reuse H, *et al*. 2001. The protein-protein interaction map of *Helicobacter pylori. Nature* **409**: 211–215.

Rindflesch TC, Hunter L, Aronson AR. 1999. Mining Molecular Binding Terminology from Biomedical Text. *Proceedings of the AMIA Annual Symposium*: 127–131.

Rindflesch TC, Tanabe L, Weinstein JN, Hunter L. 2000. EDGAR: Extraction of Drugs, Genes and Relations from the Biomedical Literature. *Pac Symp Biocomput 2000:* 515–524.

Schwikowski B, Uetz P, Fields S. 2000. A network of protein-protein interactions in yeast. *Nat Biotech* **18**: 1257–1261.

Sekimizu T, Park HS, Tsujii J. 1998. Identifying the Interaction

Copyright © 2001 John Wiley & Sons, Ltd. 

between Genes and Gene Products Based on Frequently Seen Verbs in Medline Abstracts. *Genome Inform Ser Workshop Genome Inform. 1998*; **9**: 62–71.

Stapley BJ, Benoit G. 2000. Biobibliometrics: information retrieval and visualization from co-occurrences of gene names in Medline abstracts. *Pac Symp Biocomput 2000*: 529–540.

Sussman JL, Lin D W, Jiang JS, *et al*. 1998. Protein Data Bank (PDB): database of three-dimensional structural information of biological macromolecules. *Acta Crystallogr D Biol Crystallogr* **54**: 1078–1084.

Tanabe L, Scherf U, Smith LH, Lee JK, Hunter L, Weinstein JN. 1999. MedMiner: An Internet Text-Mining Tool for Biomedical Information, with Application to Gene Expression Profiling. *BioTechniques* **27**: 1210–1217.

Thomas J, Milward D, Ouzounis C, Pulman S, Carrol M. 2000. Automatic Extraction of Protein Interactions from Scientific Abstracts. *Pac Symp Biocomput 2000*: 541–552.

Uetz P, Fields S, Rothberg JM. 2000. A comprehensive analysis of protein-protein interactions in *Saccharomyces cerevisiae*. *Nature* **403**: 623–627.

Xenarios I, Rice DW, Salwinski L, Baron MK, Marcotte EM, Eisenberg D. 2000. DIP: the Database of Interacting Proteins. *Nucleic Acids Res* **28**: 289–291.

Yakushiji A, Tateisi Y, Miyao Y, Tsujii J. 2001. Event Extraction from Biomedical Papers Using a Full Parser. *Pac Symp Biocomput 2001*: 408–419.

*The*
**Genomics**
*Website at Wiley*

**www.wiley.co.uk/genomics**

The **Genomics** website at Wiley is a new and **DYNAMIC** resource for the genomics community, offering **FREE** special feature articles and new information **EACH MONTH**.

Find out more about *Comparative and Functional Genomics*, and how to view all articles published this year **FREE OF CHARGE**!

Visit the **Library** for hot books in Genomics, Bioinformatics, Molecular Genetics and more.

Click on **Primary Research** for information on all our up-to-the minute journals, including: *Genesis*, *Bioessays*, *Gene Function and Disease*, and the *Journal of Gene Medicine*.

Let the **Genomics** website at Wiley be your **guide** to genomics-related web sites, manufacturers and suppliers, and a calendar of conferences.

WILEY

1516

Copyright © 2001 John Wiley & Sons, Ltd. 

## References

[PDF_00804] Andrade M. A., Valencia A. (1998). Automatic extraction of keywords from scientific text: application to the knowledge domain of protein families.. Bioinformatics.

[PDF_00807] Bader G. D., Donaldson I., Wolting C., Ouellette B. F., Pawson T., Hogue C. W. (2001). BIND--The Biomolecular Interaction Network Database.. Nucleic Acids Res.

[PDF_00810] Bairoch A., Apweiler R. (2000). The SWISS-PROT protein sequence database and its supplement TrEMBL in 2000.. Nucleic Acids Res.

[PDF_00812] Benson D. A., Boguski M. S., Lipman D. J., Ostell J., Ouellette B. F. (1998). GenBank.. Nucleic Acids Res.

[PDF_00817] Blaschke C., Andrade M. A., Ouzounis C., Valencia A. (1999). Automatic extraction of biological information from scientific text: protein-protein interactions.. Proc Int Conf Intell Syst Mol Biol.

[PDF_00821] Blaschke C., Oliveros J. C., Valencia A. (2001). Mining functional information associated with expression arrays.. Funct Integr Genomics.

[PDF_00824] Chien C. T., Bartel P. L., Sternglanz R., Fields S. (1991). The two-hybrid system: a method to identify and clone genes for proteins that interact with a protein of interest.. Proc Natl Acad Sci U S A.

[PDF_00828] Eilbeck K., Brass A., Paton N., Hodgman C. (1999). INTERACT: an object oriented protein-protein interaction database.. Proc Int Conf Intell Syst Mol Biol.

[PDF_00831] Eisenberg D., Marcotte E. M., Xenarios I., Yeates T. O. (2000). Protein function in the post-genomic era.. Nature.

[PDF_00833] Enright A. J., Iliopoulos I., Kyrpides N. C., Ouzounis C. A. (1999). Protein interaction maps for complete genomes based on gene fusion events.. Nature.

[PDF_00836] Fromont-Racine M., Rain J. C., Legrain P. (1997). Toward a functional analysis of the yeast genome through exhaustive two-hybrid screens.. Nat Genet.

[PDF_00839] Fukuda K., Tamura A., Tsunoda T., Takagi T. (1998). Toward information extraction: identifying protein names from biological papers.. Pac Symp Biocomput.

[PDF_00842] Hishiki T, Collier N, Nobata C, Okazaki-Ohta T, Ogata N, Sekimizu T, Steiner R, Park HS, Tsujii J (1998). Developing NLP Tools for Genome Informatics: An Information Extraction Perspective.. Genome Inform Ser Workshop Genome Inform.

[PDF_00851] Ito T., Tashiro K., Muta S., Ozawa R., Chiba T., Nishizawa M., Yamamoto K., Kuhara S., Sakaki Y. (2000). Toward a protein-protein interaction map of the budding yeast: A comprehensive system to examine two-hybrid interactions in all possible combinations between the yeast proteins.. Proc Natl Acad Sci U S A.

[PDF_00848] Jenssen T. K., Laegreid A., Komorowski J., Hovig E. (2001). A literature network of human genes for high-throughput analysis of gene expression.. Nat Genet.

[PDF_00858] Ohta Y., Yamamoto Y., Okazaki T., Uchiyama I., Takagi T. (1997). Automatic construction of knowledge base from biological papers.. Proc Int Conf Intell Syst Mol Biol.

[PDF_00865] Proux D., Rechenmann F., Julliard L. (2000). A pragmatic information extraction strategy for gathering data on genetic interactions.. Proc Int Conf Intell Syst Mol Biol.

[PDF_00861] Proux D, Rechenmann F, Julliard L, Pillet V, Jacq B (1998). Detecting Gene Symbols and Names in Biological Texts: A First Step toward Pertinent Information Extraction.. Genome Inform Ser Workshop Genome Inform.

[PDF_00871] Rain J. C., Selig L., De Reuse H., Battaglia V., Reverdy C., Simon S., Lenzen G., Petel F., Wojcik J., Schächter V. (2001). The protein-protein interaction map of Helicobacter pylori.. Nature.

[PDF_00873] Rindflesch T. C., Hunter L., Aronson A. R. (1999). Mining molecular binding terminology from biomedical text.. Proc AMIA Symp.

[PDF_00879] Schwikowski B., Uetz P., Fields S. (2000). A network of protein-protein interactions in yeast.. Nat Biotechnol.

[PDF_00887] Stapley B. J., Benoit G. (2000). Biobibliometrics: information retrieval and visualization from co-occurrences of gene names in Medline abstracts.. Pac Symp Biocomput.

[PDF_00890] Sussman J. L., Lin D., Jiang J., Manning N. O., Prilusky J., Ritter O., Abola E. E. (1998). Protein Data Bank (PDB): database of three-dimensional structural information of biological macromolecules.. Acta Crystallogr D Biol Crystallogr.

[PDF_00894] Tanabe L., Scherf U., Smith L. H., Lee J. K., Hunter L., Weinstein J. N. (1999). MedMiner: an Internet text-mining tool for biomedical information, with application to gene expression profiling.. Biotechniques.

[PDF_00898] Thomas J., Milward D., Ouzounis C., Pulman S., Carroll M. (2000). Automatic extraction of protein interactions from scientific abstracts.. Pac Symp Biocomput.

[PDF_00901] Uetz P., Giot L., Cagney G., Mansfield T. A., Judson R. S., Knight J. R., Lockshon D., Narayan V., Srinivasan M., Pochart P. (2000). A comprehensive analysis of protein-protein interactions in Saccharomyces cerevisiae.. Nature.

[PDF_00904] Xenarios I., Rice D. W., Salwinski L., Baron M. K., Marcotte E. M., Eisenberg D. (2000). DIP: the database of interacting proteins.. Nucleic Acids Res.

[PDF_00907] Yakushiji A., Tateisi Y., Miyao Y., Tsujii J. (2001). Event extraction from biomedical papers using a full parser.. Pac Symp Biocomput.

